# Age, pathology and CA-125 are prognostic factors for survival in patients with brain metastases from gynaecological tumours

**DOI:** 10.1016/j.ctro.2020.05.001

**Published:** 2020-05-06

**Authors:** S.H.J. Nagtegaal, A.F.C. Hulsbergen, E.B.L. van Dorst, V.K. Kavouridis, C.A.C. Jessurun, M.L.D. Broekman, T.R. Smith, J.J.C. Verhoeff

**Affiliations:** a Department of Radiation Oncology, University Medical Center Utrecht, HP Q 00.3.11, 3508 GA, Utrecht, the Netherlands; b Computational Neuroscience Outcomes Center, Department of Neurosurgery, Brigham & Women’s Hospital, Harvard Medical School, 75 Francis Street, Boston, MA, United States; c Departments of Neurosurgery, Haaglanden Medical Center and Leiden University Medical Center, Leiden University, J11-R-83, Postbus 9600, 2300 RC Leiden, the Netherlands; d Department of Gynaecologic Oncology, University Medical Center Utrecht, HP F.05.1.26, 3508 GA, Utrecht, the Netherlands; e Department of Neurology, Massachusetts General Hospital, Mailcode: WACC 8-835, 55 Fruit Street, Boston, MA 02114, United States

**Keywords:** Brain metastasis, Gynaecological malignancies, Prognosis, Survival

## Abstract

**Background and purpose:**

Brain metastases originating from gynaecological tumours are a rare phenomenon, but have an increasing incidence due to better targeted therapies. This study aimed to identify factors that predict survival in these patients, which can be used in creating a robust prognostic tool for shared decision making.

**Materials and methods:**

We identified a consecutive cohort of 73 patients treated for gynaecological brain metastases in two tertiary institutions. Baseline demographics, pathology and serum CA-125 were included in a multivariable Cox proportional hazards model.

**Results:**

Median overall survival in our cohort was 14.4 months, with a one-year survival of 56.4% and a two-year survival of 39.1%. Thirty-eight patients (52.1%) had ovarian carcinoma as the primary malignancy. The following factors were significantly associated with survival: age (HR 1.05 per year), CA-125 (HR 1.02 par 50 U/ml), and uterine and vulvar primary tumours (when compared to ovarian carcinoma, with HRs 3.07 and 8.70). A post-hoc analysis with primary tumour site reclassified into ovary versus non-ovary showed a HR of 0.50 for ovarian primary tumour type.

**Conclusion:**

We have found that age, pathology and CA-125 are prognostic factors for survival in patients with brain metastases from gynaecological tumours. Our findings may provide a foundation for future development of prediction models, for the benefit of both patients and physicians.

## Highlights

• The largest cohort of brain metastases patients from gynaecological tumours.
• CA-125, age and primary tumour type are prognostic for survival.
• This will inform clinical practice and aid the development of new prognostic tools.

## 1 Introduction

Brain metastases (BMs) occur in 20–40% of all patients with cancer, with 8–10% having intracranial metastasis at time of diagnosis [1], [2], [3], [4], [5]. The most frequent sites of origin being lung cancer, breast cancer and melanoma. Far less common primary tumours to metastasise to the brain are gynaecological tumours. The incidence of BMs from ovarian, endometrial and cervical carcinoma are estimated to be 0.3–2.2% [6], [7]. Even rarer primary sites include vaginal and vulvar cancers, with only a handful of cases reported [8], [9]. However, due to the advent of better targeted therapies, long-term survival in patients with gynaecological tumours has improved, with the unfortunate consequence of increased incidence of BMs [10]. Additionally, advances in imaging have made early diagnosis of previously occult BMs possible, adding to the incidence [11], [12].

As is the case in all oncologic fields, survival prediction in BMs is important to inform shared decision making between physicians and patients. Several tools have been developed to predict survival for BM patients, the most predominant being the Recursive Partitioning Analysis (RPA) [13] and the Diagnosis-Specific Graded Prognostic Assessment (DS-GPA) [14]. However, no prognostic models based on such large cohorts have been created specifically for BMs from gynaecological tumours. As a first step to such a model, we set out to identify prognostic factors for survival in a large, multi-institutional retrospective cohort of patients with BMs from gynaecological tumours.

## 2 Methods

### 2.1 Patient selection and data collection

We retrospectively selected a consecutive cohort of patients who underwent radiation therapy (RT) and/or neurosurgical resection as initial treatment for BMs originating from gynaecological tumours between 2012 and 2017 in two tertiary academic medical centres in Boston, United States and Utrecht, The Netherlands.

As this study only involved retrospective chart review, the need to obtain informed consent was waived in both institutions. In the Boston hospital, Institutional Review Board approval was obtained to conduct the study. In the Dutch hospital, permission was obtained from the institutional ethics committee.

### 2.2 Data collection and outcome

Baseline data were collected from patient records. Collected data consisted of age at first treatment for BMs, number of BMs, presence of extracranial metastases, level of serum cancer antigen 125 (CA-125), Karnofsky performance status (KPS) at baseline, primary tumour type and interval between diagnosis of the primary tumour and the BMs.

The primary outcome was overall survival from the point of initial treatment for brain metastases.

### 2.3 Statistical analysis

Patterns of missing data were analysed, and when data was deemed to be missing at random, multiple imputation was performed [15], [16].

A Cox proportional hazards model was made with the following variables: age, number of BMs, presence of extracranial metastases, CA-125, KPS, primary tumour type and interval between diagnosis of the primary tumour and the BMs. In a post-hoc analysis, a Cox proportional hazards model was made comparing ovarian with non-ovarian primary tumours along with the other variables. Additionally, the dataset was split into an ovarian and non-ovarian group, and separate Cox regression models were fit for each group. Kaplan-Meier curves were used to visualize differences in survival when stratifying for CA-125 and primary tumour type. Finally, the number of brain metastases was dichotomized into 1 or ≥2, as possible treatment options between these groups differ [17].

Statistical analyses were performed with R 3.5.1 (R Foundation for Statistical Computing, Vienna, Austria) using the ‘mice’ package [18].

## 3 Results

### 3.1 Participants

In total, 73 patients were treated for BMs of gynaecological tumours and were included in analysis. In 69 (94.5%) of these patients, pathological examination of the brain lesions confirmed the diagnosis. There were missing data for CA-125 (n = 16, 21.9%) and KPS (n = 1, 1.4%), with CA-125 only being determined for BMs of ovarian origin in one of the participating centres. Baseline characteristics after imputation are presented in Table 1, and treatment characteristics are shown in Table 2**.** The entire cohort had a median survival of 14.4 months, with a one-year survival of 56.4% and a two-year survival of 39.1%.

**Table 1:** Baseline characteristics.

	Total n = 73
	**n**
Age (median; IQR)	63 (55–69)
Number of BMs (%) 1 2 3 4 ≥5	38 (52.1) 15 (20.5) 9 (12.3) 4 (5.5) 7 (4.1)
Extracranial metastases present (%)	45 (61.6)
CA125 (median; IQR)	38 (9–97)
KPS (median; IQR)	80 (80–100)
Primary tumour type (%) Ovary Cervix Uterine sarcoma Endometrium Fallopian tube Vulva	38 (52.1) 5 (6.8) 9 (12.3) 17 (23.3) 2 (2.7) 2 (2.7)
Interval primary diagnosis and BM in months (median; IQR)	32.07 (16.49–51.98)

BM = Brain metastasis; IQR = Inter quartile range; KPS = Karnofsky performance status; SRS = stereotactic radiosurgery; WBRT: whole brain radiotherapy.

**Table 2:** Treatment characteristics.

	**n**
Treatment for BMs (%)	
Surgery	9 (12.3)
RT	7 (9.6)
RT + Surgery	57 (78.1)
Type of RT (%)	
SRS	36 (49.3)
SRT	3 (4.1)
WBRT	23 (31.5)
WBRT + SRS boost	1 (1.4)
Unknown	1 (1.4)
Dose of RT (%)	
1 × 17	1 (1.4)
1 × 18	3 (4.1)
1 × 20	1 (1.4)
1 × 21	2 (2.7)
1 × 24	2 (2.7)
3 × 8	4 (5.5)
5 × 4	2 (2.7)
5 × 5	15 (20.5)
5 × 6	7 (9.6)
10 × 3	13 (17.8)
13 × 3	1 (1.4)
14 × 2.5	2 (2.7)
15 × 2.5	6 (8.2)
17 × 2	1 (1.4)
20 × 2	1 (1.4)
Unknown	3 (4.1)
Treatments for multiple BMs (%)	
All BMs resected	2 (2.7)
1 BM resected; RT for other BMs	16 (21.9)
1 BM resected; no treatment for other BMs	9 (12.3)
2 BMs resected; no treatment for other BMs	4 (5.5)
RT for all BMs	1 (1.4)
Unknown	1 (1.4)
Interval surgery and RT in days (median; IQR)	28 (22–38)

BM = Brain metastasis; HFSRT = Hypofractionated stereotactic radiotherapy; RT = Radiotherapy; SRS = Stereotactic radiosurgery; WBRT = Whole-brain radiotherapy.

### 3.2 Primary analysis

**Fig. 1:**
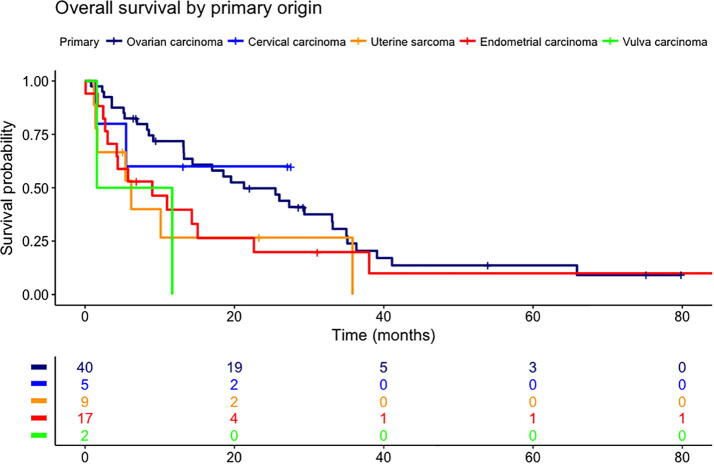
Kaplan-Meier plot comparing survival for the different primary tumour types.

**Fig. 2:**
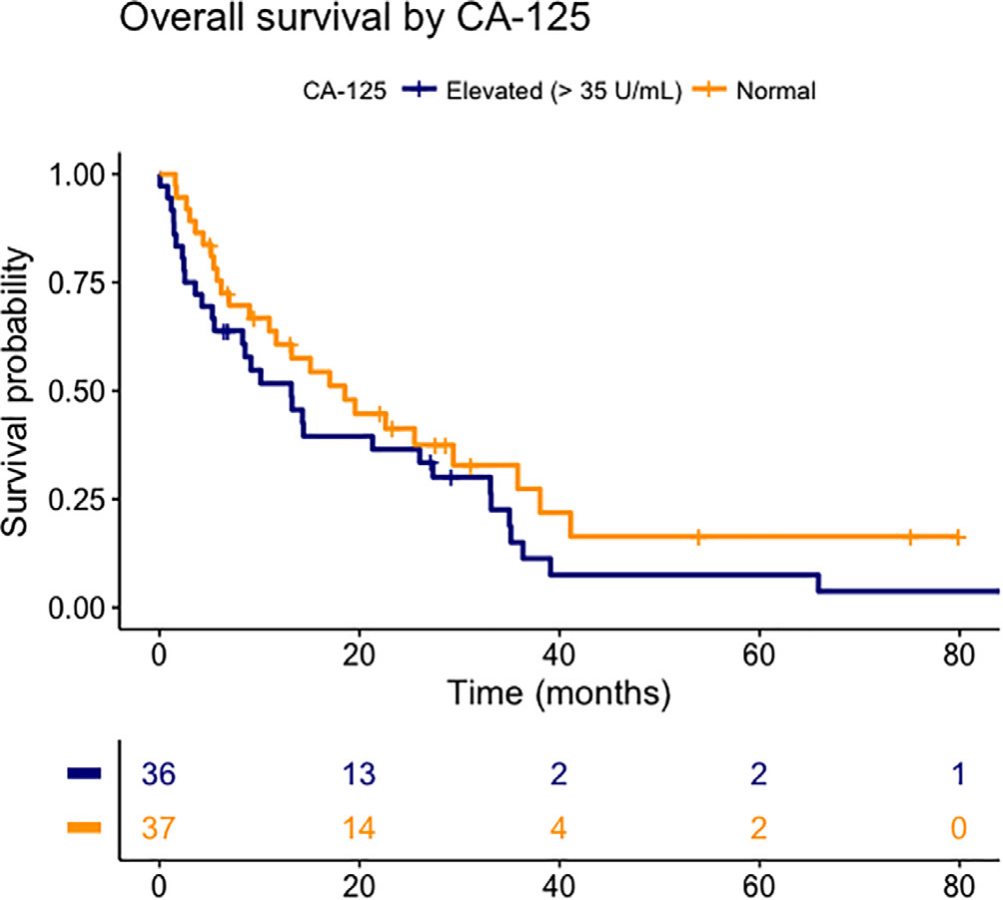
Kaplan-Meier plot comparing high and low serum CA-125 at baseline.

Results of the Cox proportional hazards model are shown in Table 3. The following factors were significantly associated with survival: age (HR 1.05 per year), CA-125 (HR 1.02 per 50 U/ml), and uterine and vulvar primary tumours (with ovarian carcinoma as a reference, with HRs 3.07 and 8.70, respectively). A Kaplan-Meier plot comparing survival for the different primary tumour types is shown in Fig. 1, and a Kaplan-Meier plot comparing high and low CA-125 values is shown in Fig. 2.

**Table 3:** Results from the Cox proportional hazards model.

Variable	HR (95% CI)	p
Age	1.05 (1.01–1.09)	**0.03**
Number of BMs	1.12 (0.97–1.29)	0.14
Extracranial metastases present	1.58 (0.85–2.95)	0.15
CA-125*	1.02 (1.01–1.03)	**<0.01**
KPS	1.00 (0.98–1.02)	0.74
Primary tumour type Ovary** Cervix Uterine sarcoma Endometrium Fallopian tube Vulva	– 1.71 (0.33–8.90) 3.07 (1.12–8.42) 1.93 (0.95–3.94) 0.51 (0.06–4.06) 8.70 (1.19–63.34)	– 0.52 **0.03** 0.07 0.52 **0.03**
Interval primary diagnosis and BM	1.00 (0.99–1.01)	0.64

BM = Brain metastases; KPS = Karnofsky Performance Status.

* In steps of 50 U/ml.

** Reference.

### 3.3 Secondary analyses

**Fig. 3:**
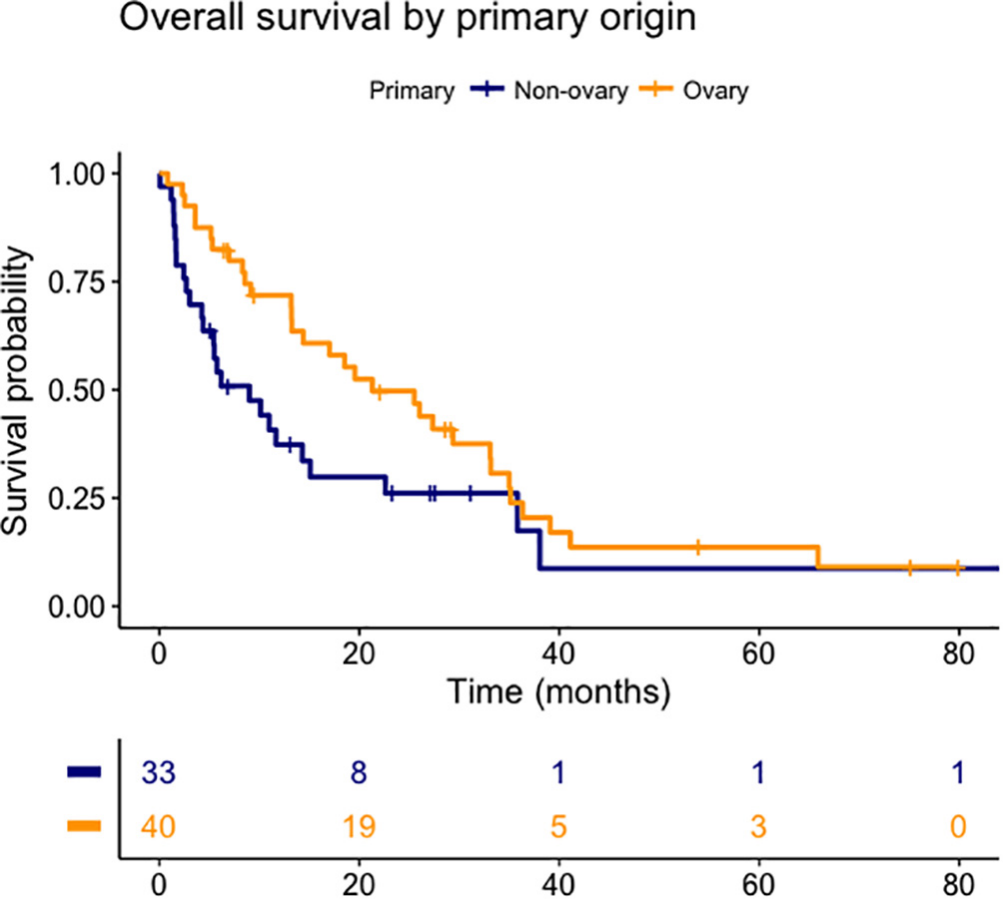
Kaplan-Meier plot comparing ovarian vs non-ovarian primary tumour (adjusted HR 0.50, CI 0.27–0.93, p = 0.03).

A post-hoc Cox model with primary tumour site reclassified into ovarian vs. non-ovarian showed that ovarian origin (HR 0.50, 95%CI 0.24–0.93, p = 0.03) and fewer BMs (HR 1.15, 95%CI 1.20–1.31, p = 0.03) were associated with improved survival. A Kaplan-Meier plot of survival comparing ovarian vs. non-ovarian primary tumour is shown in Fig. 3.

Additionally, the number of brain metastases was dichotomized into 1 or ≥2, which resulted in a significant result (HR 1.9, 95%CI 1.003–3.52, p = 0.049).

In order to assess the effect of missing data and subsequent imputation of CA-125, a sensitivity analysis was performed exclusively incorporating cases with a known CA-125 value (n = 57). This showed no changes from principal analysis, indicating that the effect of missing data was negligible. Another sensitivity analysis was performed by splitting the dataset into ovarian and non-ovarian groups. CA-125 was only significant in the ovarian subgroup (p < 0.01), with no prognostic effect seen in the non-ovarian group.

## 4 Discussion

In this study, we have identified several independent factors that are prognostic for survival in brain metastases patients with gynaecological tumours. In particular, primary tumour origin seems to be a strong predictor, along with CA-125. Additionally, in post-hoc analysis, a significant effect was found when comparing 1 and ≥2 BMs, suggesting that not the absolute number, but the solitary or multiple nature of BMs has an effect on survival.

Several other studies have attempted to identify prognostic factors in gynaecological brain metastases.

In the biggest cohort, from the MITO 19 study, 174 women with BMs from epithelial ovarian cancer were included [19]. After multivariable analysis, the following variables were significantly associated with survival: multiple BMs, extracranial metastases, age, and monotherapy.

In another cohort, Matsunaga et al. [20] presented an analysis of 70 patients undergoing Gamma Knife Surgery (GKS) for BMs from gynaecological tumours. They found significant prognostic effects for type of primary lesion, controlled extracranial disease (compared to active) and number of BMs (1 vs. ≥2). Similar to our results, KPS was not found to be significant, with and 95% CI of the HR 0.38–1.36. In a similar, smaller cohort of 33 patients undergoing GKS, Johnston et al. found age and RPA to significantly predict survival [21].

Rades et al. (n = 56) examined the prognostic value of several clinical factors in patients treated with surgery and/or radiotherapy [8]. A multivariable model showed significant prognostic value of Eastern Cooperative Oncology Group (ECOG) performance score, as well as the absence of extracranial metastases. Janssen et al. (n = 49) found a similar effect for ECOG score in a subgroup of patients who only received WBRT, but found no significant effect of extracranial metastasis [9]. Finally, Anupol et al. studied 15 patients, and found that type of therapy and the presence of extracranial metastases predict survival [22].

These results have been further used to create two nomograms by Janssen et al. and Rades et al. to predict survival [8], [9]. However, these were based on cohorts too small to make robust prediction models, and have not been validated in separate cohorts. Therefore, they have seen sparse use in clinical practise.

Serum CA-125 has been a factor of interest before in patients with gynaecological cancers who developed brain metastases. In the abovementioned study performed by Anupol et al. ten out of fifteen BM patients were found to have an elevated CA-125, although the authors found no correlation with survival [22]. Similar results have been found in other small cohorts (with sample sizes ranging from 4 to 15, see also the review by Piura et al. [23]), leading to the conclusion that serum CA-125 is not an effective biomarker for gynaecological BMs, nor a useful predictor for survival. However, the small sample sizes of these studies have limited statistical power, potentially yielding false-negative results. Our study, which is the first to investigate this variable in a large sample size, challenges these findings from previous literature.

In gynaecological practice, CA-125 is used in the diagnostic workup for ovarian cancer. On its own, the result of CA-125 testing is less reliable than other diagnostic tests like ultrasonography [24]. It has also been considered as a marker for cancer screening in the general population, but a recent review found no evidence for a beneficial effect on survival [25]. A likely reason for the limited performance of CA-125 as a diagnostic tool on its own is the fact that its level can be influenced by non-cancer related factors, like obesity, age, phase of the menstrual cycle, menopause, smoking status and history of hormone therapy [26], [27]. Aside from these issues with specificity, the sensitivity is also limited, as only 50% of patients with early stage ovarian cancer have elevated CA-125 levels. Therefore, the interpretation of CA-125 level should not be done without considering different clinical factors and imaging [28]. As a prognostic factor, however, CA-125 has shown more reliability, as it has been demonstrated to strongly predict both overall and progression free survival in patients with ovarian cancer [29]. Additionally, it has been shown to detect cancer recurrence long before symptoms occur [30]. However, the added value of repetitive post-treatment CA-125 surveillance is limited, as direct treatment at relapse detected solely by this marker (i.e. with no signs or symptoms present) does not result in a survival advantage [31]. Therefore, the clinical decision-making should not be affected by CA-125 alone, and it should be considered only as one of several prognostic factors for patients’ survival.

The observed difference in survival between ovarian and non-ovarian primary could be explained due to differing survival per tumour type, regardless of the presence of BMs. Ovarian cancer has a more favourable survival compared to other gynaecological cancers [32]. Different options with regards to targeted therapies and diagnostic methods may also influence survival.

The fact that KPS does not predict survival in our study is at odds with previous studies, which found prognostic effects of the ECOG score [8], [9]. The reason for this is not clear. It could be the case that differences in variables in the respective prognostic models causes the effect of performance status to differ between studies, as different clinical factors are corrected for. Also, the choice of performance status (KPS or ECOG) can be the cause of this discordance, as Matsunaga et al. and Johnston et al. also examined the effect of KPS, and found no significant effect [20], [21]. A final possibility is that, due to the relatively high KPS in our dataset (median 80, with an IQR of 80–100), the effects of lower KPS are harder to determine.

As with most retrospective analyses, the biggest limitation of the current study is missing data. Even though we have selected a validated way of imputing our dataset [15], [16], we cannot be sure our results would have been the same if no data were missing. However, a sensitivity analysis with only observed values shows similar results to our primary analysis, suggesting that the effect of missing data is negligible.

Similarly, other variables that could be of interest were not recorded, and could therefore not be analysed. It has been suggested that gross tumour volume could be a more predictive factor for survival than number of BMs [33]. We would have liked to explore this further, but were limited by the unfeasibility to collect these data.

Additionally, local differences in clinical protocol meant that in one centre CA-125 was only measured in patients with BMs of ovarian origin. A sensitivity analysis was done by splitting the dataset into ovarian and non-ovarian groups, which resulted in a significant prognostic effect of CA-125 in the ovarian group only. However, the sample size in the non-ovarian group was limited, suggesting there might have been insufficient power to find a significant result. Additionally, the sensitivity analysis with only observed values showed a significant predictive effect of CA-125 independent from primary tumour type.

Finally, our limited sample size limits our ability to detect smaller prognostic effects. This is an unfortunate inherency in research into rare diseases. The lack of a large number of participants also hinders our ability to create a robust and widely applicable nomogram to predict survival.

This latter point is our main suggestion for the future. Our hypothesis-generating results can be used to inform the creation of a nomogram for gynaecological BMs. This should be based on a large cohort of patients, ideally from multiple centres with different treatment patient demographics. This prediction model will aid the decision-making process and helps guide the patient and physician to the most optimal treatment, in order to provide the best possible care.

In conclusion, we have found that age, pathology and CA-125 may be prognostic factors for survival in brain metastasis from gynaecological tumours. The predictive role of CA-125 in BMs from non-ovarian origin is less clear, and remains to be further investigated. Our findings may help to inform clinical decision making, as well as identify variables of interest for the construction of robust nomograms from large, multi-institutional databases.

## Declaration of Competing Interest

The authors declare that they have no known competing financial interests or personal relationships that could have appeared to influence the work reported in this paper.
